# Response of root development and nutrient uptake of two chinese cultivars of hybrid rice to nitrogen and phosphorus fertilization in Sichuan Province, China

**DOI:** 10.1007/s11033-021-06835-7

**Published:** 2021-10-19

**Authors:** Guotao Yang, Farhan Nabi, Sumbal Sajid, Abdul Rasheed Kaleri, Ali Murad Jakhar, Liang Cheng, Martin Raspor, Noor Muhammad, Jun Ma, Yungao Hu

**Affiliations:** 1 Sichuan Agricultural University, Chengdu, 611130 Sichuan China; 2 College of Life Science and Engineering, Southwest University of Science and Technology, Mianyang, 621010 Sichuan China; 3 Institute of Plant Sciences, University of Sindh, Jamshoro, 76080 Pakistan; 4 Institute for Biological Research Siniša Stanković—National Institute of Republic of Serbia,, University of Belgrade, 11060 Belgrade, Serbia

**Keywords:** Deyou4727, Yixiangyou2115, Gene expression, Nutrient use efficiency, Root growth

## Abstract

**Background:**

Chemical fertilization helped modern agriculture in grain yield improvement to ensure food security. The response of chemical fertilization for higher hybrid rice production is highly dependent on optimal fertilization management in paddy fields. To assess such responses, in the current work we examine the yield, root growth, and expression of related genes responsible for stress metabolism of nitrogen (N) and phosphorus (P) in two hybrid-rice cultivars Deyou4727 (D47) and Yixiangyou2115 (Y21).

**Methods and results:**

The experiment followed four nitrogen (N) (N_0_, N_60_, N_120,_ and N_180_ kg/ha) and phosphorus (P) (P_0_, P_60_, P_90_, and P_120_ kg/ha) fertilizer levels. The grain yield in D47 was more sensitive to nitrogen application, while Y21 was more sensitive to phosphorus application, which resulted in comparatively higher biomass and yield. Our findings were corroborated by gene expression studies of glutamine synthetase *OsGS1;1* and *OsGS1;2* and phosphate starvation-related genes *PHR1* and *SPX*, confirming sensitivity to N and P application. The number of roots was less sensitive to nitrogen application in D47 between N_0_ and N_60_, but the overall nutrient response difference was significantly higher due to the deep rooting system as compared to Y21.

**Conclusions:**

The higher yield, high N and P use efficiency, and versatile root growth of D47 make it suitable to reduce unproductive usage of N and P from paddy fields, improving hybrid rice productivity, and environmental safety in the Sichuan basin area of China.

**Supplementary Information:**

The online version contains supplementary material available at 10.1007/s11033-021-06835-7.

## Introduction

The extensive use of chemical fertilizers in current agricultural practice is detrimental to the environment and reduces soil quality and crop production in the long run. Consequently, finding sustainable solutions for the improvement of crop production has become a challenging global agricultural issue [1]. According to FAO [2], the annual world consumption of the three main fertilizer nutrients, nitrogen (N), phosphorus (P) applied as phosphate (P_2_O_5_), and potassium applied as potash (K_2_O), reached 185 million tons (total N, P_2_O_5_, and K_2_O) in 2016 and is expected to top 200 million tons annually by 2022. As the consumption increases, it will prompt the production of commercial fertilizers, intermediates, and raw materials, which will significantly contribute to global carbon emissions. China is among the top five markets of commercial fertilizer production and consumption; according to FAO [3], China consumed 27.3 million tons N and 15 million tons P fertilizer for rice and wheat fertilization in 2014.

It has been estimated that only about 1/3 of the applied nitrogen fertilizer is used by crops, whereas 2/3 remain in the environment, accounting for ~16 billion USD worth loss of N fertilizer worldwide on annual basis [4]. Unassimilated N fertilizer leaches from farming and creates environmental damage, including nitrate accumulation, water pollution and eutrophication [5], and soil deterioration [1]. In order to avoid excessive N fertilizer usage, current research in agronomy is focused on developing strategies for enhancing the nitrogen use efficiency of crop plants [1, 4, 6–8]. In cereal crops, enhancing grain yield without increasing fertilizer input remains one of the most important challenges [9].

Problems related to the use of phosphate fertilizer include both leaching of excess phosphate into the environment [10], and the prospective depletion of finite reserves of phosphate rock, which represents the raw material for the production of fertilizer [11]. Additionally, cereals and some other food crops convert a significant part of assimilated phosphate into phytic acid, which has no nutritive value and even acts as an antinutrient [12]. Agronomic measures for the prevention of phosphate leaks into the environment have proven relatively inefficient and transgenic approaches have been suggested for improving phosphate uptake and limiting the synthesis of phytic acid for improved phosphate use efficiency [13].

Recently, an array of various N and P management strategies have been launched for gradual or spatially targeted release of commercial fertilizers [1], which include combining organic manure [14], vermicompost [15], biochar [16] and usage of nano-fertilizers for delivery of nutrients to target plants [17]. However, the use of these strategies has so far faced limitations because they are either insufficient, costly, or labor-intensive [1].

According to FAO [2] data (http://www.fao.org/faostat/en/#data/QC), rice (*Oryza sativa* L.) is the world’s third most important crop after maize and wheat, in terms of both harvested area (162 million hectares worldwide) and annual production (755 million tonnes). Furthermore, for rice fertilization in China, a 75% higher (180 kg/ha) nitrogen input is used than the world average, whereas in the Guangdong province of Southern China fertilization rates as high as 250 kg/ha are applied [18]. Among the major food crops, rice accounts for the highest amounts of wasted nitrogen fertilizer [19]. Furthermore, optimizing the right measure of phosphate fertilizer to deliver sufficient phosphorus to rice crops without risking environmental damage has proven as a major challenge for rice cultivation, for instance in West African countries [20]. More than 3/4 of the applied phosphate fertilizer is wasted and remains in the environment, as only about 1/4 is taken up by rice crops [21]. Fertilization of rice paddy fields represents an important target for decreasing large-scale consumption of commercial fertilizers, reducing the environmental damage from fertilizer use, and at the same time cutting the costs for fertilizer purchase and transport. Thus, hybrid rice varieties, which show enhanced tolerance to abiotic stresses and produce high yields in low nitrogen and phosphorus fertilization, represent a promising perspective for reducing the use of chemical fertilizers for rice cultivation [22]. Like other cereal crops hybrid rice varieties have different fertilization patterns, highly depending on their genetic nature [8, 23]. For such hybrid rice types, effective fertilization management includes selecting the proportion of fertilizer, the source of fertilizer, the timing of fertilizer application, and the combination of fertilizers that match the needs of the crop to maximize fertilizer utilization efficiency, optimize crop production, and minimize the negative impact of fertilizer on the environment [24].

This study’s main objectives were to estimate the impact of low and high nitrogen and phosphorus input on the yield, nutrient uptake, use efficiency and root growth, of hybrid rice varieties Deyou4727 and Yixiangyou2115 under different N and P levels. Also, the expression levels of N and P metabolism-related genes *OsGS1;1*, *OsGS1;2*, *PHR1*, and *SPX* were quantified to assess the molecular response of the cultivars to different nutrient concentrations. The results provide a basis for planning measures to reduce the losses of N and P in paddy fields and for choosing a well-performing variety to improve rice yield in the Sichuan Basin area of China and other regions with similar environments.

## Materials and methods

### Study area

The experiments were carried out in the laboratory and field at the Southwest University of Science and Technology in Mianyang, Sichuan Province, China, in 2019. The soil of this region is clay-loamy soil, with a bulk density of 1.29 g/cm^3^ and organic matter content of 28.6 g/kg, while total nitrogen, phosphorus, and potassium content were 1.68, 0.37, and 1.86 g/kg respectively. The major food crops cultivated in this region are rice and oilseed rape, which form a paddy-dryland rotation system in this area, with oilseed rape cultivation occurring from late September to April, and rice cultivation from April to September.

### Experimental design

Field experiment treatment sets were carried out for two rice varieties Deyou4727 (D47) and Yixiangyou2115 (Y21). Rice seeds were cultivated from March-September 2019. Field design was carried out as a randomized complete block design, each plot was 3 m × 3.5 m with 0.5 m corridors. When the seedlings were around 7-8 cm long, field plots were treated. For the N application, four N application levels were used, i.e., N_0_, N_60_, N_120_, N_180_ [kg/ha]. To ensure that nutrients other than N would not limit rice growth, 90 kg P_2_O_5_/ha was applied to each plot. For the P application, four P application levels were used, i.e., P_0_, P_60_, P_90_, P_120_ [kg/ha]. To ensure that nutrients other than P would not limit rice growth, 120 kg N/ha was applied to each plot. Each treatment was replicated three times. The plant sampling was done at four time points after treatments, wrapped in aluminum foil, frozen in liquid nitrogen, and stored at -80 °C until further analysis.

### Sampling and measurement

#### Sampling

Plant samples of all treatments were collected at four time points (10, 25, 40, 55 days after treatment) in 2019. Fresh samples were used for morphological measurements and gene expression studies. The soil samples for N and P uptake calculation were transported to the lab in ice boxes. Fresh samples were used to determine initial nutrient concentrations.

#### Measurements

At maturity, plant samples were separated into straw, leaves, and panicles for dry weight determination after oven-drying to constant weight at 70 °C. Plant roots were counted according to Gu et al., [25]. Grain yield was measured from a 5 m^2^ area from the center of each plot at the maturity stage. Panicles were placed into bags and labeled to determine yield and yield components [26]. Grains and panicles were separated. Filled and empty panicles were separated and counted, grains were weighed. The seed setting rate was calculated according to Xiang et al., [27]. Yield component data (1000-grain weight, the number of panicles, and percentage of filled grains) from all treatments were determined according to Fageria [28].

Total nitrogen content in the soil was determined by the Kjeldahl method as described by Okalebo et al. [29]. Available phosphorus was determined by the Olsen method following the procedure described by Juo [30].

### Crop response to N and P inputs

#### N and P use efficiencies

N and P use efficiency parameters were calculated based on the grain yield and N/P accumulation in plots treated at different N and P rates. The definitions are as follows [31]:

1 $${\text{Agronomic}}\,{\text{Efficiency}}\,({\text{AE}}) = \frac{{GY_{{ + N/P}} - GY_{{N/P0}} }}{{FN}}\,(kg\,grain\,kg^{{ - 1}} )$$

2 $${\text{Physiological}}\,{\text{Efficiency}}\,({\text{PE}}) = \frac{{GY_{{ + N/P}} - GY_{{N/P0}} }}{{TN/P_{{ + N/P}} - TN_{{N/P0}} }}\,(kg\,grain\,kg^{{ - 1}} )$$

3 $${\text{Recovery}}\,{\text{Efficiency}}\,({\text{RE}}) = \frac{{TN_{{ + N/P}} - TN_{{N/P0}} }}{{FN}}\, \times \,100\% \,(\% )$$

where GY_+N/P_ is the grain yield of the plots that received N and P fertilizer, GY_N/P0_ is the grain yield in the N_0_ and P_0_ plots, FN is the amount of N and P fertilizer applied, TN_+N/P_ is the total N and P accumulation in the plots that received N and P fertilizer at maturity, and TN_N/P0_ is the total N and P accumulation in the N_0_ and P_0_ plots at maturity.

### qPCR analysis

Total RNA from flag leaves of mature rice plants was isolated using the RNeasy Plant Mini Kit (Qiagen, Venlo, The Netherlands) and reversely transcribed using Omniscript RT Kit (Qiagen). Quantitative real-time PCR (qPCR) was performed using the SYBR Premix Ex Taq (TaKaRa, Dalian, China) and the CFX96^TM^ Real-Time PCR Detection System (Bio-Rad, Hercules, CA, USA). The PCR mix was composed of 5 µl SYBR Premix Ex Taq, cDNA corresponding to 30 ng RNA, 0.3 µl of each F and R primer (10 mM), and PCR grade water up to the final volume of 10 µl. The primer sequences are available in Supplementary Table S1. The incubation temperature of the reaction was as follows: (1) denaturation at a cycle of 95 °C for 3 min; (2) amplification: 40 cycles at 95 °C for 30 s, 60 °C for 30 s, 72 °C for 30 s; (3) final elongation 72 °C for 3 min, and (4) melting curve analysis (65–95 °C). Each sample was analyzed in triplicate, and the relative expression levels were calculated relative to control (N_0_ or P_0_ treatment) using the 2^−ΔΔCt^ comparative CT method [32].

### Statistical analysis

Origin 19.0 and Excel 2016 statistical software were used to analyze the experimental data. The correlation analysis between grain yield and N/P accumulation was done using ggpubr R package. Each treatment value is expressed as mean ± standard deviation (SD) of 3-6 biological replicates. The differences were determined by Student’s *t*-test at *P* < 0.05 (*), 0.01 (**), or 0.001 (***).

## Results

### Yield and physiological traits responses

The effect of nutrient and genotype on hybrid rice grain yield and effective panicles was significant (*P* < 0.05). Both rice cultivars Deyou4727 (D47) and Yixiangyou2115 (Y21) showed a maximum yield of rice grains in high nitrogen (N) treatment N_180_ and the lowest yield observed in no-N treatment N_0_. The cultivar D47 showed a maximum yield of 9.17 t/ha, in the high N treatment N_180_, which was 5% higher than for Y21. The response of both cultivars to N application was significantly different. D47 was more sensitive to N application as compared to Y21, as it showed a significantly higher difference of yield response between N_0_–N_60_, N_60_–N_120_, N_120_–N_180_ (Table 1). Yield response was positively correlated with effective panicles in D47 and Y21, which resulted in yield difference. Y21 was less sensitive to N application.

**Table 1:** Effect of N and P fertilizer on the rice yield parameters for two rice varieties Deyou4727 **(**D47) and Yixiangyou2115 **(**Y21)

	D47					Y21				
	Yield (t/ha)	EP (grain/panicle)	SSR (%)	GPP (grain/panicle)	1000-grain weight (g)	Yield (t/ha)	EP (grain/panicle)	SSR (%)	GPP (grain/panicle)	1000-grain weight (g)
N_0_	6.32d	7.89d	0.92b	150.27b	35.43a	6.15b	7.29c	0.93a	134.24c	34.04b
N_60_	7.00c	8.57c	0.94a	156.15b	36.18a	6.80b	7.65c	0.94a	140.76b	34.22b
N_120_	8.45b	9.88b	0.95a	163.13ab	36.41a	8.15a	8.17b	0.95a	151.36a	34.86a
N_180_	9.17a	11.05a	0.93a	170.90a	35.74a	8.75a	9.48a	0.91b	158.85a	35.41a
P_0_	6.62c	7.96c	0.91b	157.07b	35.14a	6.21b	7.83c	0.92b	142.43b	33.84b
P_60_	7.57b	8.98a	0.92b	162.04a	35.90a	6.84b	8.15b	0.94a	147.31b	34.20b
P_90_	8.45a	9.88a	0.95a	163.13ab	36.41a	8.15a	8.17b	0.95a	151.36a	34.86a
P_120_	7.80b	9.15a	0.95a	161.11a	35.89a	7.74ab	8.48a	0.93a	146.77b	34.57ab

*EP* effective panicle, *SSR* seed setting rate, *GPP* grains per panicle. Values obtained at different N, or different P treatments, at the same time point for the same cultivar, and labeled with different letters are significantly different from each other at *P* < 0.05

The rice type D47 overall showed significantly higher grain yield in all treatments, more specifically under different nitrogen treatments it showed 3%, 3%, 4%, and 5% higher grain yield in N_0_, N_60_, N_120_, and N_180_ treatments respectively, as compared to Y21.

Among different phosphorus (P) treatments, both rice types favored the moderate phosphorus concentration P_90_. Likewise, in different phosphorus treatments, D47 showed, 6%, 10%, 4%, and 1% higher grain yield than Y21 in P_0_, P_60_, P_90,_ and P_120_ treatments respectively. The response of P application for both cultivar yields were different. D47 showed higher sensitivity of yield response from P_0_–P_60_, P_60_–P_90_ and P_90_–P_120_, as compared to Y21, which showed maximum sensitivity between P_60_-P_90_. The P application response was equally observed for effective panicles that resulted in yield differences respectively.

Similarly, effective panicle significantly increased with increasing N and P treatments. Higher nutrient input caused higher effective panicles in both rice types, and they followed a N_180_ > N_120_ > N_60_ > N_0_, and P_120_ > P_90_ > P_60_ > P_0_ trend, for nitrogen and phosphorus respectively. Unlike EP, seed setting rate, the number of grains per panicle, and 1000-grain-weight were less affected by N and P supply (Table 1).

### Correlation analyses of grain yield and N/P accumulation

A Pearson’s correlation was computed to assess the relationship between grain yield and nutrient accumulation for both rice types (Fig. S1). Mostly negative correlations between low or high nutrient accumulation and yield were found in different nitrogen and phosphorus treatments. A high increase in nutrient uptake was negatively correlated with the amount of yield output. More specifically, in D47 in N_180_ treatment, was positively correlated (R = 0.69) while in Y21 N_60_ and N_120_ were more positively correlated with R values of (R = 0.76) and (R = 1) respectively.

Similarly in the case of phosphorus accumulation D47 showed a positive correlation in all treatments up to P_120_. The correlation was highest upto P_60_ (R = 1), while it slightly decreased in P_60_ and P_90_ with R values near 0. 94 and 0.97 respectively. Unlike D47, Y21 showed a negative correlation (R = – 0.76), (R = – 0.94) at P_60_ and P_90_ respectively, however, it had a good positive correlation at P_120_ with R-value near 0. 99.

### Plant biomass and nutrient accumulation in hybrid rice

Total biomass at maturity significantly (*P* < 0.05) increased with N application rate and differed in two hybrid rice cultivars (Table 2). The highest plant biomass, N, and P accumulation were found in high N and P treatments for both hybrid rice varieties. Overall D47 showed 4%, 9%, 3%, and 6% higher biomass production than Y21 in N_0_, N_60_, N_120_, and N_180_ treatments respectively. A similar trend was found for different P treatments, in which D47 showed 10%, 7%, 3%, and 7% higher biomass production than Y21 in P_0_, P_60_, P_90,_ and P_120_ treatments respectively. Like biomass, N and P also followed higher accumulation in high treatments and low accumulation in low treatments. N accumulation followed the N_180_ > N_120_ > N_60_ > N_0_ trend, and phosphorus accumulation followed the P_120_ > P_90_ > P_60_ > P_0_ trend in both rice types, at constant environmental factors. D47 accumulated 5–10 % greater total biomass compared to Y21 at all fertilizer treatments (Table 2). Accumulation of nitrogen biomass was similar for both cultivars at all treatments, however, D47 accumulated 10–15 % more phosphorus biomass on most treatments (Table 2).

**Table 2:** Biomass, N, P content, and accumulation for two rice varieties Deyou4727 (D47) and Yixiangyou2115 (Y21)

Treatments	D47			Y21		
	N/P accumulation (g m^2^)	Biomass (g m^2^)	N/P concentration (%)	N/P accumulation (g m^2^)	Biomass (g m^2^)	N/P concentration (%)
N_0_	7.7d	1325d	0.58b	7.5d	1277c	0.59b
N_60_	9.9c	1572c	0.63b	9.8c	1424b	0.69a
N_120_	12.4b	1713b	0.73a	12.2b	1663a	0.73a
N_180_	14.1a	1848a	0.76a	13.8a	1738a	0.79a
P_0_	2.68c	1249d	0.21a	2.21b	1119c	0.19b
P_60_	3.15b	1488c	0.21a	2.84b	1378b	0.20b
P_90_	3.66b	1713b	0.21a	3.24a	1663a	0.19b
P_120_	4.19a	1817a	0.23a	3.82a	1698a	0.22a

Values obtained at different N, or different P treatments, at the same time point for the same cultivar, and labeled with different letters are significantly different from each other at *P* < 0.05

N/P accumulation, biomass and N/P concentration in both cultivars were sensitive to N and P application. Like yield and yield parameters, N/P accumulation response and biomass were higher in D47. It showed a more sensitive response between N_0_–N_60_, N_60_–N_120_, N_120_–N_180_. Unlike N/P accumulation and biomass, D47 showed less sensitivity in N/P concentration. That makes it more nitrogen efficient, as compared to Y21. Conversely, Y21 showed less response sensitivity in biomass and N/P concentration, while N/P accumulation was significantly more sensitive between N_0_–N_60_, N_60_–N_120_, N_120_–N_180_.

### Nutrient use efficiency

Nutrient uptake and nutrient use efficiency varied among treatments and hybrid rice varieties. The AE, PE, and RE significantly (*P* < 0.05) increased with nutrient application, thus the highest values were found in N_120_ and P_90_ treatments for both hybrid rice types (Table 3). The AE for N_120_ treatment in D47 showed 6%, 65%, and 2% higher AE, PE, and RE respectively as compared to Y21. Unlike N_120_, in the case of P_90_, Y21 showed 6%, 15%, and 5% higher AE, PE, and RE in P_90_ treatment as compared to D47. The response of nutrient use efficiency of both cultivars was highly sensitive to N application and showed a significant difference of AE and PE. A more sensitive response was found between N_60_–N_120,_ while Y21 showed less sensitivity for these values as compared to D47. In case of NRE most sensitive difference was found between N_120_–N_180_ for both cultivars, with the less significant difference (*P <*0.05). Similarly, the P application resulted in a high response difference of AE and PE between P_60_–P_90_ for both cultivars, however, D47 was more sensitive for P application as compared to Y21. When it comes to RE, D47 showed a high response difference between P_60_–P_90_ while Y21 showed more response difference between P_90_–P_120_ and no significant difference found between P_60_–P_90_. These findings suggest that D47 had a more sensitive response difference for N and P applications in each treatment, while Y21 was less sensitive to nutrient application. The above results indicate higher nitrogen use efficiency in D47.

**Table 3:** AE, PE, RE for two rice varieties Deyou4727 (D47) and Yixiangyou2115 (Y21)

Treatments	D47			Y21		
	AE (kg grain kg^−1^)	PE (kg grain kg^−1^)	RE (%)	AE (kg grain kg^−1^)	PE (kg grain kg^−1^)	RE (%)
N_0_	–	–	–	–	–	–
N_60_	11.31c	30.33c	37.31b	10.85c	14.04c	38.56a
N_120_	17.75a	92.10a	39.48a	16.68a	31.35b	38.61a
N_180_	15.85b	60.22b	35.47c	14.47b	41.39a	34.96b
P_0_	–	–	–	–	–	–
P_60_	15.81b	35.31b	7.81c	10.46c	28.39c	10.60b
P_90_	20.32a	57.95a	10.85b	21.54a	68.08a	11.45b
P_120_	9.83c	32.20c	12.56a	12.77b	47.27b	13.47a

*AE* agronomic efficiency, *PE* physiological efficiency, *RE* recovery efficiency. Values obtained at different N, or different P treatments, at the same time point for the same cultivar, and labeled with different letters are significantly different from each other at *P* < 0.05

### Impact of nutrient input on root number

**Fig. 1:**
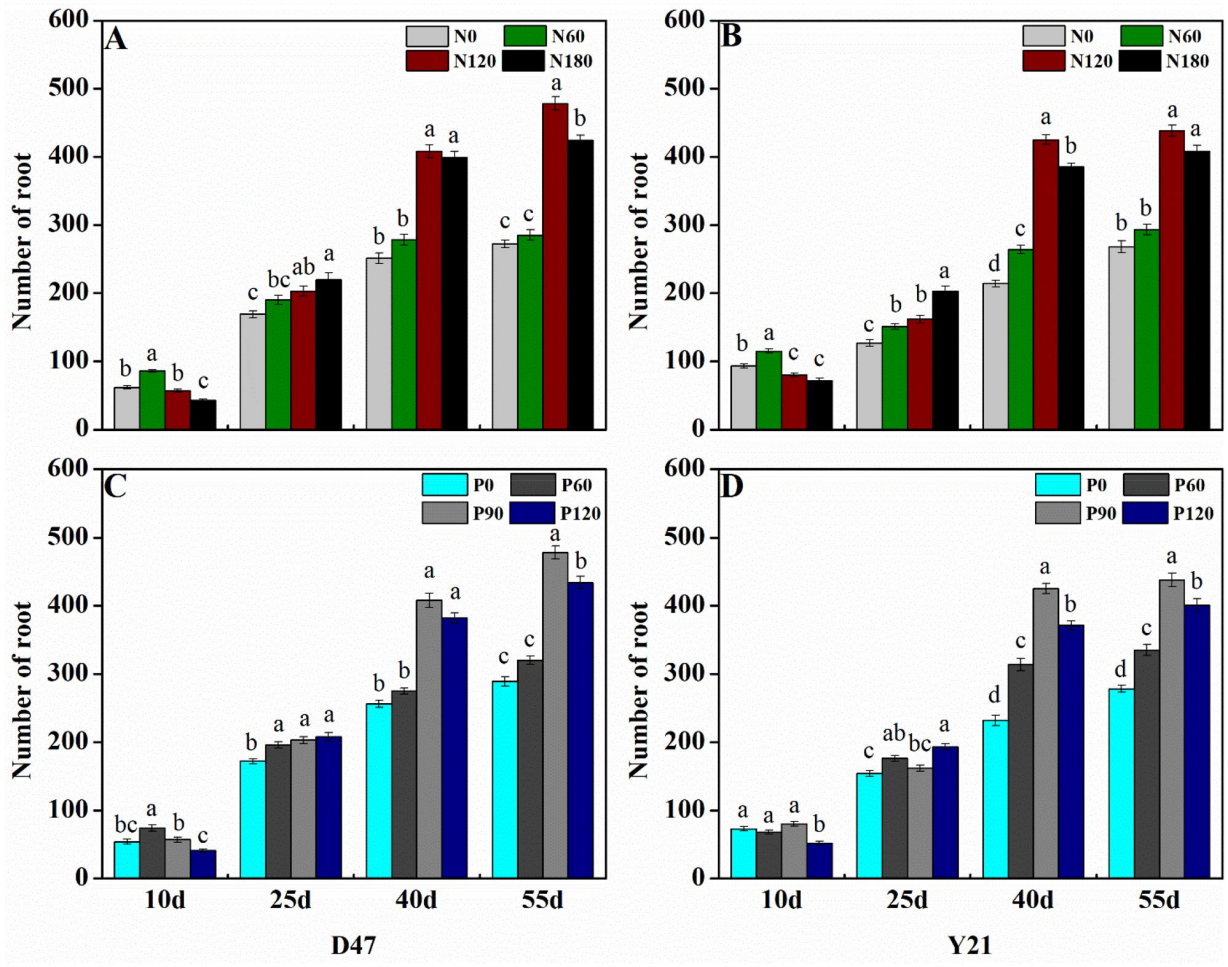
Root number in Deyou4727 (D47) (**A**) and Yixiangyou2115 (Y21) (**B**) at different nitrogen treatments, While Deyou4727 (D47) (**C**) and Yixiangyou2115 (Y21) (**D**) at different phosphorus treatments from day 10 to 55 after fertilization. Values obtained at different N, or different P treatments, at the same time point for the same cultivar, and labeled with different letters are significantly different from each other at *P < *0.05

The number of roots increased progressively from day 10 to day 55 in all treatments, but the number of roots and dynamics of root appearance differed between treatments (Fig. 1), which indicates nutrient (N, P) application had a low response difference in initial days. Among the nitrogen treatments, the highest number of roots on day 55 was recorded in N_120_ (Fig. 1a, b). Similarly, in the case of different phosphorus concentrations, D47 and Y21 favored the P_90_ phosphorus concentration (Fig. 1a, b). As compared with Y21, D47 collectively showed 12.4% higher root numbers in N_120_ P_90_ (478 for D47, as compared to 425 for Y21). D47 showed better development of root architecture and higher root numbers in all treatments of N and P, as compared to Y21 (Fig. S2). For N application higher sensitive difference was found between N_60_-N_120_ for both cultivars, while in the case of P application higher sensitive difference was found between P_60_-P_120_ in both cultivars. However, D47 was less sensitive in low nitrogen application N_0_–N_60_, but the overall nutrient response difference was significantly higher due to the deep rooting system as compared to Y21.

### Expression of glutamine synthetase genes *OsGS1;1* and *OsGS1;2* with nitrogen fertilization

The expression levels of two genes of the glutamine synthetase family, *OsGS1;1* and *OsGS1;2*, which are considered important markers of nitrogen metabolism, have been analyzed in response to nitrogen availability in hybrid rice cultivars D47 and Y21.

**Fig. 2:**
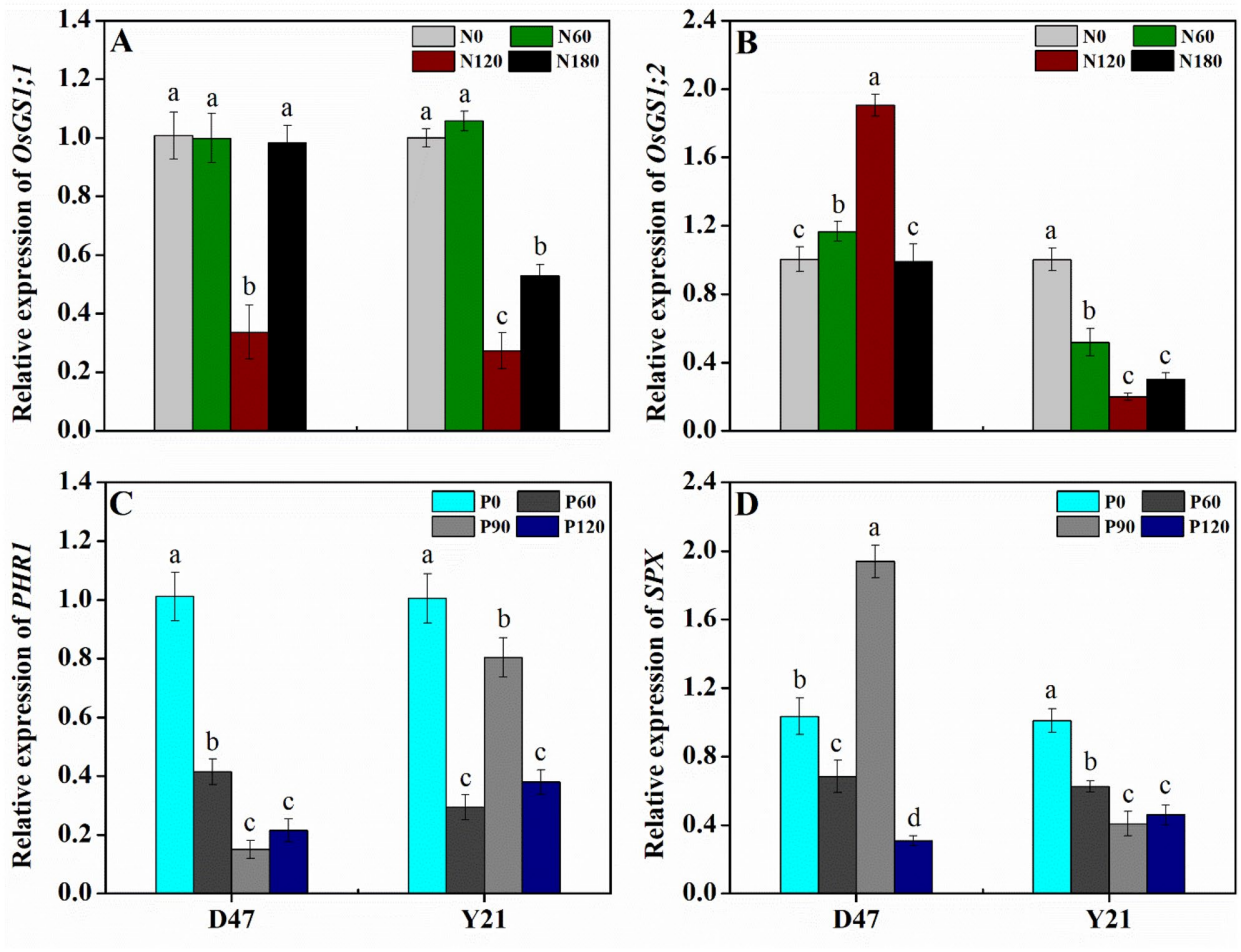
Expression levels of *OsGS1;1* (**A**) and *OsGS1;2* (**B**) genes in different nitrogen treatments, while *PHR1* (**C**) and *SPX* (**D**) genes in different phosphorus treatments in rice cultivars Deyou4727 (D47) and Yixiangyou2115 (Y21). Values obtained at different treatments within the same cultivar, and labeled with different letters are significantly different from each other at *P < *0.05

Interestingly, *OsGS1;1* was upregulated in both low (N_0_, N_60_) and high (N_180_) nitrogen compared to moderate nitrogen treatment (N_120_), where its expression in both cultivars was relatively low (Fig. 2a). The expression of *OsGS1;2* followed the opposite pattern in D47, being strongly upregulated on moderate nitrogen treatment compared to low and high nitrogen treatments. However, in Y21, *OsGS1;2* was upregulated at low nitrogen and downregulated on moderate and high nitrogen (Fig. 2b).

### Expression of *PHR1* and *SPX* genes with phosphorus fertilization

The expression levels of two genes that are considered important markers of phosphorus availability, *PHR1*, and *SPX*, have been analyzed in response to phosphorus availability in hybrid rice cultivars D47 and Y21.

The gene *PHR1* was strongly upregulated on the no-phosphorus (P_0_) treatment compared to the other three treatments for the cultivar D47, while in Y21 its expression was also higher on moderate (P_90_) than on high (P_120_) phosphorus treatment (Fig. 2c). On the other hand, the expression of *SPX* was remarkably upregulated at P_90_ compared to other treatments, while in Y21 it was downregulated on higher P concentrations compared to low P treatments (Fig. 2d).

## Discussion

Optimization of fertilizer dosage has become one of the most important challenges in the current agronomy of cereal crops [33, 34]. This work represents a comparative analysis of the effects of high, moderate, low-dosage application and non-application of nitrogen and phosphorus fertilizer on the growth, yield, yield-related traits and expression of selected genes in two Chinese hybrid rice cultivars. The cultivar Deyou4727 (herein shortened to D47) has been previously described as drought-tolerant, with a particularly well-developed root system [35], while Yixiangyou2115 (referred to as Y21) was reported as relatively nitrogen-inefficient compared to other hybrid rice cultivars and restorer lines [36].

### Yield and yield-related parameters and correlation analysis

Our results showed that grain yield progressively increased with nitrogen fertilization in both cultivars, although in Y21 the yield difference between N_120_ and N_180_ was not statistically significant (Table 1). The underperformance of Y21 with high nitrogen fertilization is in concordance with its low nitrogen efficiency as compared to other rice cultivars, as has been previously reported by Li et al. [36], and demonstrated further by comparing the nitrogen use efficiency of the two cultivars in this study.

Both cultivars performed better at moderate (P_90_) than at high (P_120_) phosphorus fertilization (Table 1). It was reported before that high phosphorus fertilization is inefficient in promoting the agronomic performance of rice, as no significant differences in grain yield were found between phosphorus applied at 90 and 135 kg/ha in both upland and paddy rice [37].

Effective panicle was positively affected by both high N and P fertilization in both cultivars, but neither high N nor high P fertilization could significantly enhance the seed-setting rate, the number of grains per panicle, or 1000-grain weight in cultivars (Table 1). Overall, it can be concluded that while high N fertilizer dosage contributed to higher yield in D47 (but not Y21), a high dosage of P fertilizer does not confer any significant benefit to the most important agronomic parameters of either D47 or Y21 output.

Hybrid rice varieties possess different nutrient uptake abilities, the low and high N and P application rate determines plant physiological needs. In our study, we assessed 2 rice types for yield and nutrient uptake correlation. We found D47 had a positive correlation with N application only in N_180_ treatment (R = 0.69), while Y21 on the other hand had a positive correlation at N_60_ and N_120_ with R-values of 0.76 and 1 in respective treatments. In our study D47 findings are positively consistent with [38], in which they found increasing N application positively correlated yield uptake up to N_180_, but in the case of Y21, the results were different. It showed a positive correlation of R=1 in the N_120_ treatment. Similarly, many studies have shown that rice yield increases with the increase of nitrogen application within a certain range, but the yield decreases when the nitrogen application is too high [39, 40].

In the case of P accumulation and yield correlation, the hybrid variety D47 showed a positive correlation up to P_120_. The correlation was highest up to P_60_ (R = 1), while it slightly decreased in P_90_ and P_120_ with R-values near 0.94 and 0. 97 respectively. Unlike D47, Y21 showed a negative correlation at P_60_ and P_90_, however, it had a positive correlation at P_120_ with R-value near 0. 99. The above findings suggest that D47 is a more phosphorus efficient type and its yield highly depends on phosphorus input, while in the case of Y21 the low P input resulted in a negative correlation, which makes it less phosphorus efficient type. Similar results were stated by Zhang et al. [37], in which they reported that P accumulation highly depends on rice variety used and P input. High input resulted in high yield (positive correlation), while between varieties the P accumulation and yield output differed greatly.

### Nitrogen and phosphorus uptake and use efficiency

In our study, high N and P fertilization positively affected both total plant biomass, and accumulation of N or P, respectively, in the plant biomass (Table 2). It is well known that N is the limiting nutrient for plant growth because it is at the same time both largely required for the synthesis of proteins and nucleic acids, and limitedly available to the plants [19]. High nitrogen fertilization is known to positively affect total biomass in rice, as well as height, grain harvest index, and components related to grain yield [38, 41–43]. Phosphorus fertilization up to 90 kg/ha positively affected plant growth, yield, formation of panicles, tillers and leaves in hybrid rice [44], whereas increasing P fertilization over that threshold was not found agronomically useful in previous reports [37].

The genotypes D47 and Y21 differed in N and P uptake (Table 2) and use efficiency (Table 3) at different fertilizer dosages. The genotype D47 was superior (~5–10 %) at total biomass compared to Y21 at all fertilizer treatments (Table 2). It showed similar N uptake rates as Y21 (Table 2) but 1.5-3-fold higher nitrogen use efficiency on all fertilizer treatments (Table 3). It is already known that nitrogen use efficiency can considerably vary between lowland rice genotypes [41]. Superior nitrogen use efficiency of D47 compared to Y21 is likely accounting for its more successful total biomass accumulation, as these two parameters are known to greatly coincide [45].

When it comes to phosphate assimilation, D47 accumulated slightly higher phosphate biomass than Y21, but this was probably only because of its greater total biomass, as its percentage of phosphorus biomass was similar to Y21 (Table 2). However, Y21 showed considerably higher values for phosphorus use efficiency compared to D47 on all P fertilizer treatments, especially for physiological P use efficiency (Table 3). Similar to nitrogen, phosphate use efficiency has also been proven to considerably vary between lowland rice genotypes, and it also importantly correlates with rice grain yield [46].

Both nitrogen and phosphate use efficiency are reported to have relatively low values in rice – NUE typically ranges from 30 to 50% [4] while PUE is typically less than 30% [47]. Therefore, breeding the rice varieties with improved nutrient use efficiency is the most promising strategy to raise both grain yield and nutritional quality with limited fertilizer input [48]. The nutrient use efficiency parameters such as agronomic use efficiency (AE), physiological use efficiency (PE), and recovery efficiency (RE) were studied in this experiment. AE, PE, and RE significantly (*P* < 0.05) increased with the nutrient application, but both AE and PE dropped at the highest fertilizer dosage of both nitrogen and phosphate. An important exception is the PE value for nitrogen fertilization of the Y21 cultivar, which was extremely nitrogen-inefficient at low and moderate doses and continued to grow even at N_180_ (Table 3). We conclude that the values of nitrogen and phosphate use efficiency of rice cultivars D47 and Y21 argue in favor of moderate fertilizer application to achieve better nutrient use efficiency.

### Root growth of D47 and Y21 genotypes at different fertilizer treatments

In our findings, root growth depended on both genotype, and fertilizer dosage (Fig. 1). In most treatments, root growth was more pronounced in D47, which is a cultivar known for drought tolerance and a well-developed root system [35]. It has been suggested before, that root growth is strongly related to nitrogen use efficiency in cereals [49, 50] so it is unsurprising that these two traits are co-occurring in the same genotype, D47.

Both nitrogen and phosphorus fertilization had a positive impact on root growth up to moderate doses (N_120_, or P_90_), whereas high fertilizer doses of either N or P (N_180_, or P_120_, respectively) had an inhibitory effect on root growth, resulting in significantly lesser number of roots compared to moderate doses starting from day 40 (Fig. 1). Ammonium toxicity from excess N fertilization can negatively affect root growth [51, 52]. Furthermore, on day 10, both D47 and Y21 had developed a greater number of roots when grown at N_60_ compared to N_120_ and N_180_ treatments, whereas since day 25 this relationship was reversed to favor higher doses of N fertilizer application (Fig. 1). It has been recently reported that urea from the N fertilizers inhibits the early stages of root growth, but does not affect shoot growth [8]. When it comes to the effect of phosphorus fertilizer on root growth, it has been shown that the number of roots in upland rice is positively affected by growing amounts of P fertilizer up to a certain limit [53], which was confirmed by our results.

### Expression of nitrogen and phosphate homeostasis-related genes

The need for improving the nutrient use efficiency of large-scale cultivated crops has pushed the research towards the identification of target genes for genetic modifications [54].

The key step in nitrogen assimilation in higher plants is the incorporation of the inorganic ammonium ion (NH_4_^+^) into organic compounds. The initial step in this pathway is the synthesis of the amino acid glutamine through the activity of the enzymes glutamine synthetases (GS) [55]. Thus, the genes encoding glutamine synthetases are important genetic markers for nitrogen assimilation and were proven related to the nitrogen use efficiency in crop species such as maize [56] and tobacco [57]. The glutamine synthetase gene family in plants consists of several genes coding for different enzyme isoforms that are differentially regulated [58]. In this study, we investigated the expression of two cytosolic GS1 (*OsGS1;1* and *OsGS1;2*) genes in rice cultivars D47 and Y21 to assess their role in response to different N fertilization treatments. The two GS1 genes have different roles in rice, with *OsGS1;1* being responsible for the initiation of synthesis of a broader range of metabolites [59, 60], whereas *OsGS1;2* has diverse functions, like the primary assimilation of ammonium ions in roots [61] and cross-talk with other signaling pathways, like cytokinins which affect the outgrowth of axillary buds in the rice shoot [62]. Previous research has shown that expression profiles of *OsGS1;1* and *OsGS1;2* at different N fertilizer treatments can vary between rice cultivars, with expression peaks coinciding with optimal levels of N fertilization for each cultivar [63]. Our results for *OsGS1;2* expressions suggest that moderate N levels (N_120_) are optimal for the cultivar D47, whereas in Y21 which is a less nitrogen-efficient cultivar, *OsGS1;2* has relatively lower expression and is more active at lower doses of nitrogen fertilization (Fig. 2).

Members of the PHOSPHATE STARVATION RESPONSE (*PHR*) gene family have been identified as MYB transcription factors with a regulatory role in response to phosphorus nutrient deficiency [64, 65]. The *PHR1* family has 12 members in rice, designated *OsPHR1*-*OsPHR12* [66]. The role in phosphate homeostasis was confirmed for *OsPHR2* [67, 68] and *OsPHR4* [69]. Our results show that in both D47 and Y21 *OsPHR1* has high transcriptional activity when P fertilizer is not applied, whereas their activity is downregulated with the growing application of P fertilization (Fig. 2), confirming its role in regulating plant metabolism at low P nutrition.

Members of the *PHR* family are subject to negative regulation by members of another family of transcription factors, *SPX* [45, 70]. The *SPX* genes might have a role in a broader array of physiological processes, but just like *PHR*, they might serve as genetic targets for improving phosphorus use efficiency [45]. The expression profiles of the *OsSPX* gene studied in our work revealed a similar pattern as for *OsPHR*, with high transcript levels when P fertilizer is not applied, but being downregulated at higher P treatments. Expression levels for both *OsPHR* and *OsSPX* were similar for both cultivars D47 and Y21 (Fig. 2).

## Conclusions

In this research, we investigated an array of agronomic traits related to yield and nutrient use efficiency of the rice cultivars Deyou4727 (D47) and Yixiangyou2115 (Y21) at low, moderate and high levels of nitrogen and phosphorus fertilization. D47 was more sensitive to nitrogen application, the response of total biomass and yield was greater than Y21, but Y21 was more sensitive to phosphorus application. We confirmed our outcomes by gene expression studies of glutamine synthetase *OsGS1;1* and *OsGS1;2* and phosphate starvation-related genes *PHR1* and *SPX*, which confirmed the D47 response sensitivity to N application and Y21 response sensitivity to P application.

Similarly, root numbers of D47 were less sensitive in low nitrogen application N_0_ and N_60_, but the overall nutrient response difference was significantly higher due to the deep rooting system as compared to Y21. Our results suggest that large amounts of N fertilizer have limited benefits for D47, whereas P fertilization with more than 90 kg/ha brings no significant agronomic benefit for the cultivation of D47 or Y21 rice. We further recommend research should be undertaken in the area of optimal fertilization management based on hybrid rice type, soil properties, and regional conditions to solve agronomic needs and environmental concerns.

## Supplementary Information

Below is the link to the electronic supplementary material.

Supplementary file1 (DOCX 1227 kb)
