# Supplementary material for: Response of root development and nutrient uptake of two chinese cultivars of hybrid rice to nitrogen and phosphorus fertilization in Sichuan Province, China
Source: Mol Biol Rep. 2021 Oct 19;48(12):8009–21. doi: 10.1007/s11033-021-06835-7 (PMC8604849; doi:10.1007/s11033-021-06835-7)
Supplement: Supplementary file 1 — Supplementary file1 (DOCX 1227 kb) [file 11033_2021_6835_MOESM1_ESM.docx]

**Supplementary data**


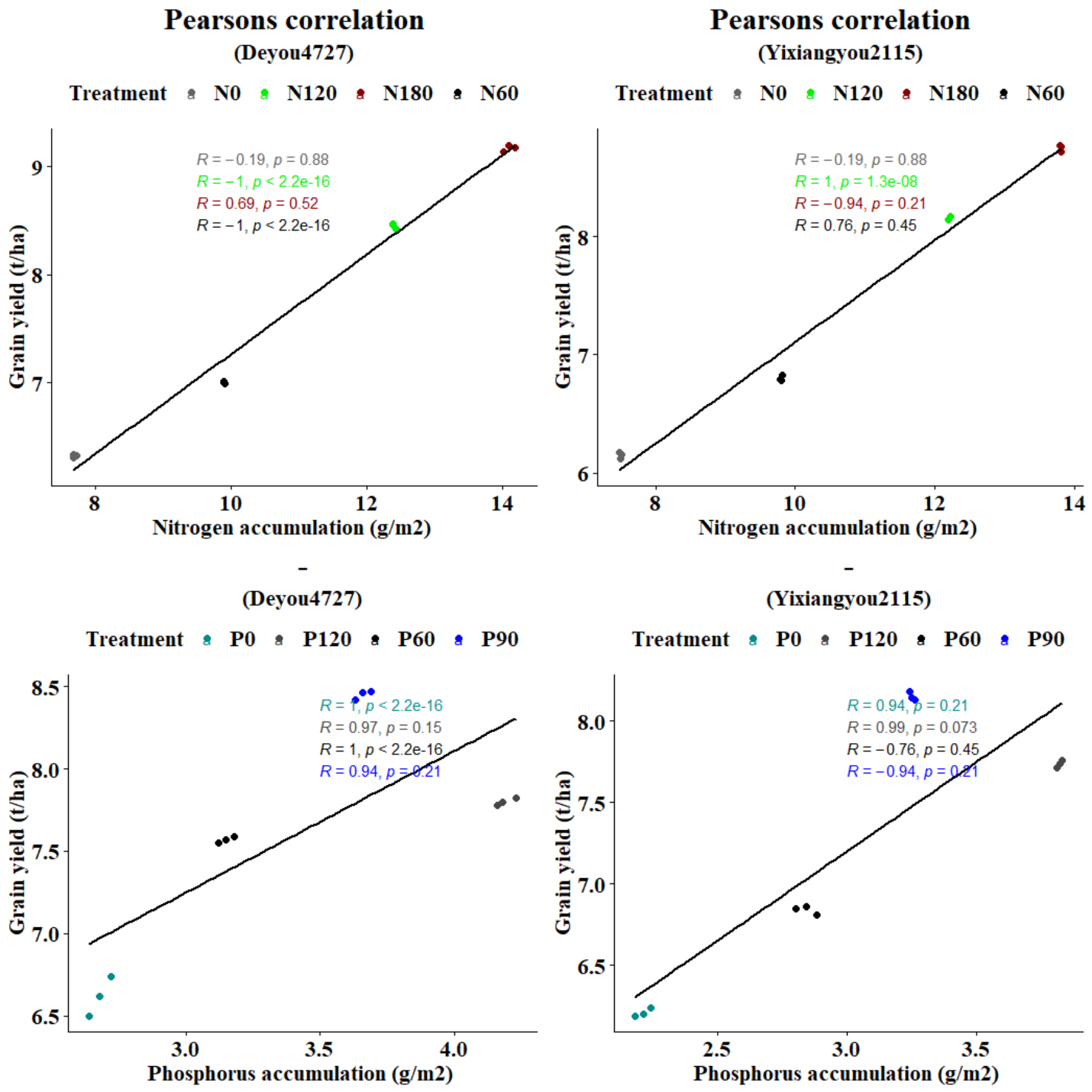


**Fig. S1** Pearson’s correlation between yield and N/P accumulation of two-hybrid D47 and Y21 on different N/P treatments, **(A)** Deyou4727 and **(B)** Yixiangyou2115 showed different treatments of nitrogen, while **(C)** Deyou4727 and **(D)** Yixiangyou2115 showed different treatments of phosphorus.


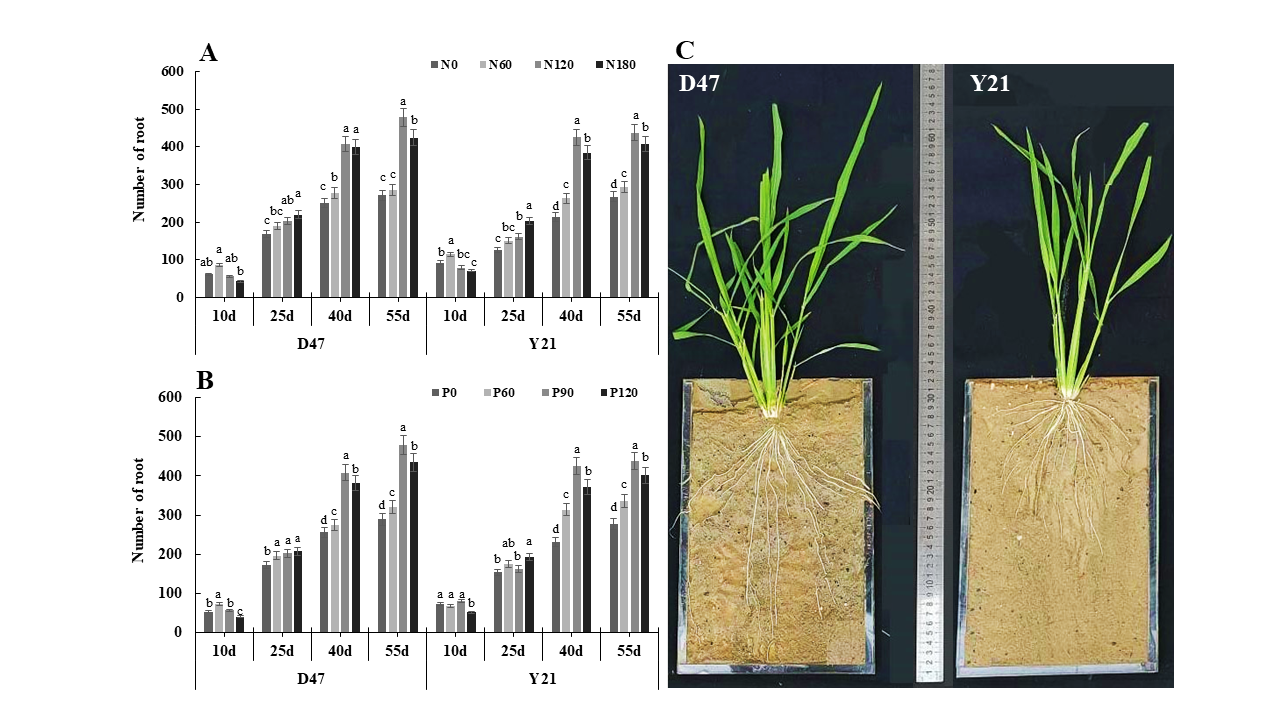


**Fig. S2** Comparative root structure of both rice cultivars Deyou4727 (D47) and Yixiangyou2115 (Y21)

**Table S1.** The primer sequences used for qRT-PCR verification

| **Gene ID** | **Function Annotation** | **Forward primer** | **Reverse Primer** |
| --- | --- | --- | --- |
| Os01g0239000 | PHR1 | 5'-CGCAAGGTGAAGGTGGACT-3' | 5'-CGATGTTGTGGCGAGTAG-3' |
| Os07g0614700 | SPX | 5'-CCCATCCAATGACCACC-3' | 5'-TTGAAAGCCAAAACACG-3' |
| Os02g0735200 | OsGS1.1 | 5'-CAAGTCTTTTGGGCGTGATATTGTTGAC-3' | 5'-CACCTGATCACCGGCAGAAATGCCGACA-3' |
| Os03g0223400 | OsGS1.2 | 5'-AAAGGCGTTCGGCCGCGACATCGTGGAC-3' | 5'-CACTTGGTCAGCAGCGGCGATGCCAACT-3' |
